# Exploring the Risk Factors for Oral Cancer in Pakistan: A Systematic Literature Review

**DOI:** 10.3390/dj12020025

**Published:** 2024-01-29

**Authors:** Muhammad Feroz Khan, Richard P. Hayhoe, Russell Kabir

**Affiliations:** School of Allied Health, Faculty of Health, Medicine and Social Care, Anglia Ruskin University, Chelmsford CM11SQ, UK; kmuhammadferoz@gmail.com (M.F.K.); richard.hayhoe@aru.ac.uk (R.P.H.)

**Keywords:** oral cancer, systematic review, Pakistan, risk factors

## Abstract

Background: Oral cancer is one of the major public health issues in Pakistan and is the second most common malignancy in the country. This is mainly attributed to the widespread use of smokeless tobacco products, cigarettes, and paan without tobacco. This review aims to go beyond commonly discussed factors and, consequently, to provide a comprehensive picture of all the multi-faceted contributors to the high prevalence of the carcinoma of the oral cavity, including the role of human papillomavirus and genetic predisposition. The aim is to synthesise all available evidence on the predisposing factors of oral carcinoma in Pakistan. Methods: This is a comprehensive systematic review of all observational studies investigating the contributing factors of malignancy of the oral cavity in Pakistan, and it strictly follows the PRISMA guidelines. Multiple databases, such as PubMed, EBSCO CINAHL Plus, SCOPUS, and Ovid Medline, were used to find studies, and the Cochrane Database of Systematic Reviews was searched for existing/ongoing reviews carried out on the same topic. A meta-synthesis of selected studies was carried out to create robust and statistically valid conclusions. Results: ST and cigarette smoking were found to be the major contributors to the burden of carcinoma of the lip and oral cavity. The included studies indicated that genetic predisposition and human papillomavirus could be major risk factors for the disease in the Pakistani population, but not enough research has been carried out to find their true impact. Conclusions: Smokeless tobacco, cigarette smoking, genetic predisposition, and human papillomavirus can be considered significant risk factors for oral cancer in Pakistan.

## 1. Background

The term oral cancer has been defined as any malignancy of the mucosal lip, tongue, gum, oral cavity floor, and palate [1]. It is a significant public health issue globally, but there is a notable disparity in its prevalence. In 2018, an estimated 177,384 deaths and 354,864 new cases of lip and oral cavity cancers were reported, with Asia alone accounting for over 70% of the deaths [2]. During the same year, 159,750 new cases and 98,851 deaths were reported in South-Central Asia by the International Agency for Research on Cancer [3]. Pakistan is one of the chief hubs of this disease; and along with China and India, it ranks amongst the top three countries with the highest incidence of OC [4].

It is the second most common cancer in the country after breast cancer, forming 10.58% of the total burden. For males, it is the most common malignancy and accounts for 15.89% of cases. In 2020, it was reported as the second-leading cause of death and was responsible for 11.27% of all cancer-related deaths in Pakistan [5]. Thus, it is one of the most urgent public health problems in the country and requires immediate attention.

The population’s high susceptibility to the disease is attributed to the widespread availability and consumption of smokeless tobacco formulations such as betel nut, gutka, naswar, and chalia [6]. The common misconception that chewing tobacco is safer compared to smoking is a contributing factor to the tobacco epidemic in the country, even though the harmful effects of chewing tobacco on human health are well-documented [7].

In addition, there are many other causes for the high prevalence of OC, such as the presence of Human Papillomavirus [8] and socioeconomic factors [9]. Cultural taboos, smoking tobacco, and genetic predisposition also play a significant role [10].

No comprehensive review outlining all the important risk factors for OC in Pakistan has been conducted in the past. Considering its alarmingly high prevalence, the authors believe that conducting a systematic review (SR) of the available literature is an essential step in understanding and countering its high incidence. This review looks beyond commonly discussed risks such as tobacco use and extends to lesser-known contributing factors such as HPV infection, genetic predisposition, and socioeconomic factors. By critically appraising all the available data on the topic, this SR will further identify gaps in research and provide valuable insights for future reviewers, general physicians, and dental surgeons. It can also provide a guiding framework for any public health workers working on decreasing the OC burden in Pakistan. The aim of this review is to identify, analyse, and synthesise all known risk factors associated with oral cancer in Pakistan.

## 2. Methods

### 2.1. Research Question

This SR will synthesise and analyse all available evidence on risk factors for oral cancer in Pakistan.

### 2.2. Study Design

This review has included cross-sectional, case–control, and descriptive studies investigating the risk factors for oral cancer in Pakistan.

### 2.3. Search Strategy

Following guidelines given by the Centre for Reviews and Dissemination 2009 [11], a preliminary review of the available literature was performed to justify the requirement for this SR. PubMed, EMBASE, and EBSCO CINAHL Plus databases were utilised for this purpose. Following that, a search was conducted in the Cochrane Database of Systematic Reviews (CDSR) to identify any pre-existing or currently ongoing systematic reviews. Different reviews were found related to OC and its risk factors, but there has been no such review conducted for Pakistan. Google Scholar was employed to search for non-indexed research articles.

The Preferred Reporting Items for Systematic Reviews and Meta-Analysis (PRISMA) guidelines were followed, and an exhaustive literature search was conducted to locate publications. The systematic review was listed in a public registry (International Platform of Registered Systematic Review and Meta-analysis Protocols) under reference number INPLASY2023120107. This search was not constrained to a timeframe or location within Pakistan, and multiple databases were utilised to avoid missing important publications and to minimise bias. The databases used for this study were PubMed, EBSCO CINAHL Plus, SCOPUS, Ovid MEDLINE, and Ovid EMBASE. The PECOS search strategy (Population, Exposure, Comparison, Outcome, and Study Design) indicated the eligibility criteria (Table 1).

The following keywords were employed to search the databases in combination: ‘oral cancer’, ‘oral carcinoma’, ‘malignancy in oral cavity’, ‘carcinoma of the lip and oral cavity’, ‘lip carcinoma’, ‘oral squamous cell carcinoma’, ‘risk factors’, ‘contributing factors’, ‘predisposing factors’, and ‘Pakistan’. Boolean operators (AND, OR) were used to refine the search strategy. RefWorks software 2023 was used for indexing and recording all articles. The reference lists of included studies and relevant grey literature were screened.

Search limits were employed to limit the scope of the search. The search was limited to primary peer-reviewed articles exclusively published in English. Furthermore, the reference lists of relevant studies were examined through the database search to identify additional studies.

**Figure 1:**
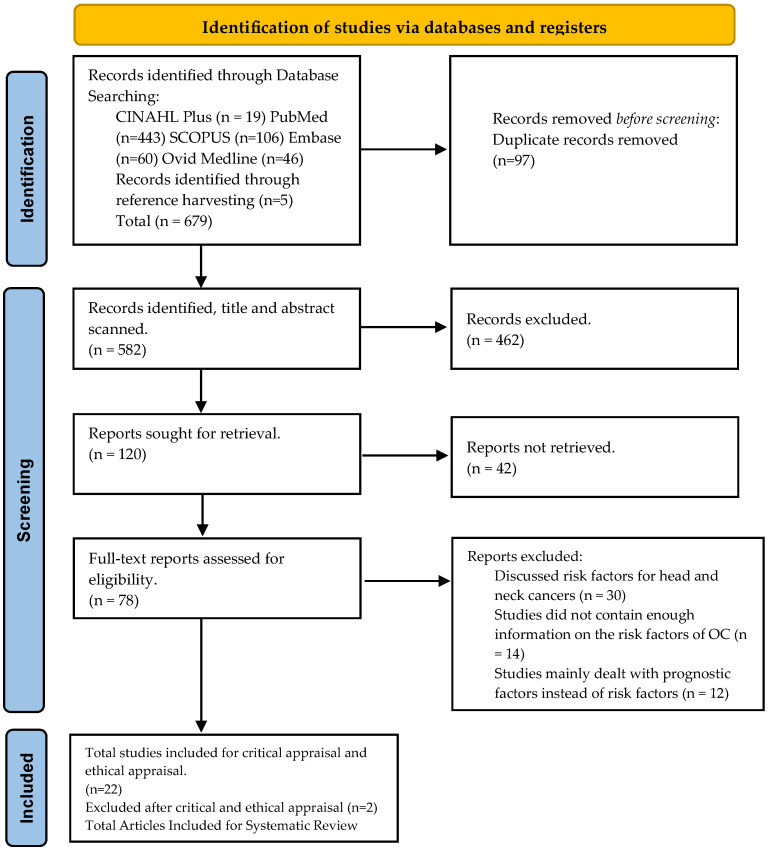
PRISMA Chart.

The database search resulted in 674 results, while 5 additional results were obtained from reference harvesting (Figure 1).

### 2.4. Study Selection

The inclusion and exclusion criteria are listed in Table 2.

Before implementing the eligibility criteria, duplicated articles were removed using RefWorks, following which manual screening was performed. After removing all duplicates, the search yielded a total of 584 articles. For higher quality research and minimising bias, only peer-reviewed articles were included.

### 2.5. Implementation of Eligibility Criteria

Initially, research papers obtained from the study search were screened and 97 duplicates were excluded. Next, the titles and abstracts of the search results were scanned and topics obviously irrelevant to the scope of the study were removed. Secondary research studies and systematic reviews were also excluded.

Following this, full-text articles were retrieved wherever possible, and 42 articles had to be excluded because full articles could not be retrieved. The articles retrieved were then screened for eligibility and the studies where risk factors for head and neck cancers were discussed as a whole were excluded. Some articles dealt mainly with prognostic factors and did not contain sufficient information on the risk factors for OC in Pakistan to warrant inclusion (Figure 1).

After the implementation of the eligibility criteria, 22 research articles were finalised for the critical and ethical appraisal stage.

### 2.6. Data Extraction and Analysis

Relevant data were extracted from each research article and recorded using Microsoft Excel for Microsoft 365 MSO (Version 2312). For all the studies, the in-text citations of the research, design, and the context in which it was conducted, e.g., area, and size of the sample, were included. Key variables, including demographic characteristics, lifestyle factors, socioeconomic factors, genetic predisposition, and exposure to risk factors such as tobacco, alcohol, human papillomavirus, and other environmental agents, were systematically extracted and tabulated. This comprehensive data extraction process aimed to provide the evidence to demonstrate the multifaceted interplay between all the risk factors and incidences of OC.

This review contains data from qualitative research only, hence meta-synthesis was conducted. Following the use of Microsoft Excel for the organisation and analysis of all important data, a textual narrative synthesis of all the important themes was conducted.

### 2.7. Critical Appraisal

The 22 selected studies were critically appraised to determine their specific merits/strengths, limitations, validity, as well as biases. In addition, this helped determine whether the studies were designed and executed reliably and whether they gave meaningful answers related to the research question. Three appraisal tools were utilised.

For case–control studies, the Critical Appraisal Skills Program (CASP) was used to refine statistical analysis (Table 3). In this program, the articles were appraised on three broad areas, namely validity, actual results, and relevance of results to the study being conducted.

For cross-sectional studies, the appraisal tool for cross-sectional studies (AXIS) was utilised. This consists of a 20-point questionnaire that addresses many important factors, such as study quality, validity, and reporting (Table 4 and Table 5).

There is no specific tool for the critical appraisal of descriptive retrospective studies, so the JBI Critical Appraisal Checklist for Analytical Cross-Sectional Studies was used for these as it was the most appropriate to judge the validity and reliability of the studies (Table 6).

Ethical appraisal was included in this review to improve ethical quality by excluding and avoiding any articles with obvious ethical inadequacies.

### 2.8. Outcome of the Critical Appraisal

In total, 20 studies were included in the final review following the appraisal, and two studies were removed due to the limited validity and reliability of the results [12,13].

**Table 3:** Critical appraisal for qualitative studies using the Critical Appraisal Skills Programme (CASP) tool.

Case–Control Studies: CASP Tool	Section A: Are the Results of the Trial Valid?							Section B: What Are the Results?			Section C: Will the Results Help Locally?	
References	Did the Study Address a Clearly Focused Issue?	Did the Authors Use an Appropriate Method to Answer the Question?	Were the Cases Recruited in an Acceptable Way?	Were the Controls Selected in an Acceptable Way?	Was the Exposure Accurately Measured to Minimise Bias?	Aside from the Experimental Intervention, Were the Groups Treated Equally?	Have the Authors Taken Account of the Potential Confounding Factors in the Design and/or Their Analysis?	How Large Was the Treatment Effect?	How Precise Was the Estimate of the Treatment Effect?	Do You Believe the Results?	Can the Results be Applied to Local Population?	Do the Results of This Study Fit with Other Available Evidence?
Mugheri et al. (2018) [14]	+	+	+	+	+	+/-	-	Outcome significantly affected by exposure	Authors considered all important variables, a relatively narrow confidence interval indicating high precision	+	+	+
Awan et al. (2016) [15]	+	+	+	+	+	+	-	Exposure was highly significant for developing the outcome	Relatively narrow confidence intervals indicating high precision	+	+	+
Zakiullah et al. (2015) [10]	+	+	+	+	+	+	+	Exposures show association with the outcome	Narrow confidence intervals indicating high precision	+	+	+
Masood et al. (2011) [16]	+	+	+	+	+	+	+	Increased risk associated with exposure	Narrow confidence interval indicating high precision	+	+	+
Zil-e-Rubab et al. (2018) [17]	+	+	+	+	+	+	+	Increased risk associated with exposure	Narrow confidence interval indicating high precision	+	+	-
Sarwar et al. (2022) [18]	+	+	+	+	+	+	+	Increased risk associated with exposure	Narrow confidence interval indicating high precision	+	+	+
Khan et al. (2017) [19]	+	+	+	+	+	+	+	Increased risk associated with exposure	Wide confidence interval indicating low precision	+	+	+
Merchant et al. (2000) [20]	+	+	+	+	+	+	+	Outcome strongly associated with exposure	Relatively narrow confidence interval indicating high precision	+	+	+
Shahid et al. (2019) [21]	+	+	+	+	+	+	+	Outcome strongly associated with exposure	+/-	+	+	+
Azhar et al. (2018) [22]	+	+	+	+	+	+	+	Increased risk associated with exposure	+/-	+	+	+
Khan et al. (2020) [23]	+	+	+	+	+	+	+	Increased risk associated with exposure	Relatively wide confidence interval indicating low precision	+	+	+
Mehdi et al. (2019) [24]	+	+	+	+	+	+	+	Outcome strongly affected by exposure	Narrow confidence interval indicating high precision	+	+	+
Aqeel et al. (2017) [12]	-	+/-	+	+	+	+	-	Outcome significantly affected by exposure	No information about CI or *p*-value was given	+/-	+/-	+
Alamgir et al. (2022) [25]	+	+	+	+	+	+	+	Outcome affected by exposure	Some of the results were statistically insignificant, and varying degrees of precision were observed	+	+	+
Alamgir et al. (2022) [26]	+	+	+	+	+	+	+	Outcome affected by exposure in some cases	Moderate degree of precision indicated by an acceptable confidence interval	+	+	+
Alamgir et al. (2021) [27]	+	+	+	+	+	+	+	Outcome affected by exposure	High degree of precision indicated by a low confidence interval	+	+	+

(+) = item adequately addressed, (-) = item not adequately addressed, (+/-) = item partially addressed.

**Table 4:** Critical appraisal for cross-sectional studies using the Appraisal tool for Cross-Sectional Studies (AXIS) Part 1.

Reference	Introduction	Methods									
	Were the Aims/ Objectives of the Study Clear?	Was the Study Design Appropriate for the Stated Aim(s)?	Was the Sample Size Justified?	Was the Target/ Reference Population Clearly Defined? (Is It Clear Who the Research Was about?)	Was the Sample Frame Taken from an Appropriate Population Base So That It Closely Represented the Target/ Reference Population under Investigation?	Was the Selection Process Likely to Select Subjects/ Participants that Were Representative of the Target/ Reference Population under Investigation?	Were Measures Undertaken to Address and Categorise Non-Responders?	Were the Risk Factor and Outcome Variables Measured Appropriate to the Aims of the Study?	Were the Risk Factor and Outcome Variables Measured Correctly Using Instruments/ Measurements That Had Been Trialled, Piloted, or Published Previously?	Is It Clear What Was Used to Determine Statistical Significance and/or Precision Estimates? (e.g., *p*-Values and Confidence Intervals)	Were the Methods (Including Statistical Methods) Sufficiently Described to Enable Them to Be Repeated?
Yasin et al. (2022) [13]	+	+	-	+	-	-	NA	+	+	+	+
Naqvi et al. (2020) [28]	+	+	-	+	+	+	NA	+	+	+	+
Mohiuddin et al. (2016) [29]	+	+	+	+	+	+	+	+	+	+	+

(+) = item adequately addressed, (-) = item not adequately addressed.

**Table 5:** Critical appraisal for cross-sectional studies using the Appraisal tool for Cross-Sectional Studies (AXIS) Part 2.

Reference	Results					Discussion		Others	
	Were the Basic Data Adequately Described?	Does the Response Rate Raise Concerns about Non-Response Bias?	If Appropriate, Was Information about Non-Responders Described?	Were the Results Internally Consistent?	Were the Results Presented for All the Analyses Described in the Methods?	Were the Authors’ Discussions and Conclusions Justified by the Results?	Were the Limitations of the Study Discussed?	Were There Any Funding Sources or Conflicts of Interest that May Affect the Authors’ Interpretation of the Results?	Was Ethical Approval or Consent of Participants Attained?
Yasin et al. (2022) [13]	+	NA	NA	+	+	+	+	-	+
Naqvi et al. (2020) [28]	+	N/A	NA	+	+	+	+	+	+
Mohiuddin et al. (2016) [29]	+	N/A	N/A	+	+	+	+	-	+/-

(+) = item adequately addressed, (-) = item not adequately addressed, (+/-) = item partially addressed.

**Table 6:** JBI Critical Appraisal Checklist for Analytical Cross-Sectional Studies.

	Were the Criteria for Inclusion in the Sample Clearly Defined?	Were the Study Subjects and the Setting Described in Detail?	Was the Exposure Measured in a Valid and Reliable Way?	Were Objective and Standard Criteria Used for Measurement of the Condition?	Were Confounding Factors Identified?	Were Strategies to Deal with Confounding Factors Stated?	Were the Outcomes Measured in a Valid and Reliable Way?	Was Appropriate Statistical Analysis Used?
Alamgir et al. (2016) [30]	+	+	+	+	-	-	+	+
Baig et al. (2012) [31]	+	+	+	+	-	-	+	+
Junaid et al. (2019) [32]	+	+	+	+	-	-	+	+

(+) = item adequately addressed, (-) = item not adequately addressed.

## 3. Results

### 3.1. Main Features of Studies Included

This review included 20 studies conducted in Pakistan. In total, 13 [15,17,20,21,22,24,25,26,27,28,29,30,31] of the studies were conducted in Karachi, whereas 7 [10,14,16,18,19,23,32] were carried out in different cities such as Peshawar, Islamabad, Hyderabad, and Lahore. For six of the included studies, the main aim was not to explore any or all risk factors for oral cancer; however, they collected adequate information on the topic to warrant inclusion.

### 3.2. Designs of Included Studies

All the 20 studies included were observational/qualitative in design, out of which 14 were case–control studies and three were retrospective studies. Furthermore, three hospital-based cross-sectional studies were included. A summary of the characteristics, designs, and findings of the studies included in this review is presented in Table 7.

#### 3.2.1. Use of Smokeless Tobacco Products Is the Most Important Contributing Factor

This review reveals that smokeless tobacco use is the most significant contributor to OC in Pakistan, and several studies confirmed their widespread use and dangers. Awan et al. [15] found that the use of gutka (betel quid) held the highest risk of developing OC among all tobacco-related products. Similarly, those using other ST products i.e., Supari and naswar, were at a four times greater risk compared to non-users. Mohiuddin et al. [29] supported these findings, reporting a significant association between the consumption of betel quid with tobacco and malignant transformation.

Junaid et al. [32] reported that out of 200 subjects with confirmed malignancy of the oral cavity, 22.5% reported being habitual naswar users. In Khyber Pakhtunkhwa, as reported by [19], naswar use was a contributor to about 70% of the cases. In one study [27], it was observed that ST use was more strongly associated with OC cases compared to cigarette smoking.

Merchant et al. [20] found that individuals consuming paan without tobacco had almost 10-fold greater chances of developing malignancy of the oral cavity as opposed to those who did not use the product, indicating that this may independently cause the malignancy in some cases.

#### 3.2.2. The Role of Smoking

Junaid et al. [32] observed tobacco smoking to be significantly associated with an increased frequency of oral cancers (*p*-value = 0.000). Out of 200 patients with proven malignancy, 61.5% of them were reported to be smokers. Khan et al. [19] reported that ever smokers had a two-fold increased risk of developing OC compared to non-smokers.

One study conducted in Hyderabad and adjoining areas of Sindh [14] discovered that the combination of smoking and alcohol constituted a high risk for OC.

Azhar et al. [22] reported a notable trend in smokers. Among participants who smoked exclusively without any other contributing habit, only 23.8% were cases and 76.2 percent were controls. However, respondents who smoked cigarettes in conjunction with other contributing habits (11.3%) constituted 92.8% of the cases and a mere 7.2% of the controls.

#### 3.2.3. Genetic Predisposition to Developing OC

Several studies included in this review report that certain individuals are more prone to developing OC as opposed to others who may report similar threatening habits. Alamgir et al. [25] reported that when homozygous CYP1AQ and null GSTM1 variants are present simultaneously in the same individual, the risk of developing OC significantly increases. The risk was significantly higher than when any of these genotypes were present individually. Another study conducted by Alamgir et al. [26] found that the distribution of tobacco-metabolising enzymes varied amongst populations of different ethnicities.

A case–control study conducted by Zakiullah et al. [10] observed that individuals with both GSTM1 and GSTT1 genes had a three-fold greater risk of developing oral malignancies. Additionally, individuals with all three known polymorphisms had a 16-fold higher risk of oral cancer.

Another study conducted by Masood et al. [16] in Islamabad discovered that alterations in CYP1A1, GSTM1, GSTT1, and GSTP1 genotypes could be considered a significant factor in developing the malignancy. Variations in genetic polymorphisms of these genes, along with changes in their expression and functionality, can potentially lead to an increase or decrease in the activation of carcinogens and their subsequent detoxification, ultimately resulting in variations in OC risk.

Furthermore, Mehdi et al. [24] observed a notable correlation between the CC genotype of survivin and the risk of developing oral squamous cell carcinoma (OSCC).

#### 3.2.4. HPV Contributes to the OC Burden

Human Papillomavirus (HPV) is a known contributing factor to OC [8]. Most included studies found a strong correlation between the presence of HPV and the development of OSCC. Patients infected with HPV 16/18 had significantly greater chances of developing the malignancy as opposed to patients who did not have this virus. Those who had both viruses simultaneously in their oral rinse possessed a still greater risk [17].

One descriptive study conducted in Karachi reported that the presence of HPV was 2.7% greater in gutka chewers for over 10 years as opposed to those consuming the product for less than a decade [31].

Sarwar et al. [18] established a strong association between high-risk HPV infection and patients with OC, and 48% of the patients have detectable HPV. It also reported that tobacco use, poor oral hygiene, and HPV infection are important environmental factors that can act synergistically in the modulation of the expression of NF-kB pathway proteins.

A study conducted by Naqvi et al. [28] is in contrast with most studies: HPV was absent in all subjects, and CMV was detected in a mere 5%, whereas 25.86% were EBV-positive.

#### 3.2.5. The Role of Socioeconomic and Cultural Factors

Most included studies did not explore the role that socioeconomic and cultural factors may play in the distribution of oral cancer, which indicates that more studies need to be conducted that take a holistic approach.

Mugheri et al. [14] found that a lower standard of living (lower income and tough working conditions) may predispose individuals to the use of harmful causative factors such as the use of tobacco and alcohol, and most patients suffering from OC were uneducated, underweight, and working as labourers. Alamgir et al. [27] found that the mean age of patients with oral cancer was in the fifth decade of life, which indicates that age, in addition to long-term use of tobacco, could be a contributing factor. Cultural factors play a significant role in the distribution of OC; for instance, studies conducted in Khyber Pukhtoonkhwa [19,32] indicate that the use of naswar is a major causative factor in the region, whereas studies conducted in Karachi [15,30] found gutka chewing to be the major factor contributing to the high burden of OC.

**Figure 2:**
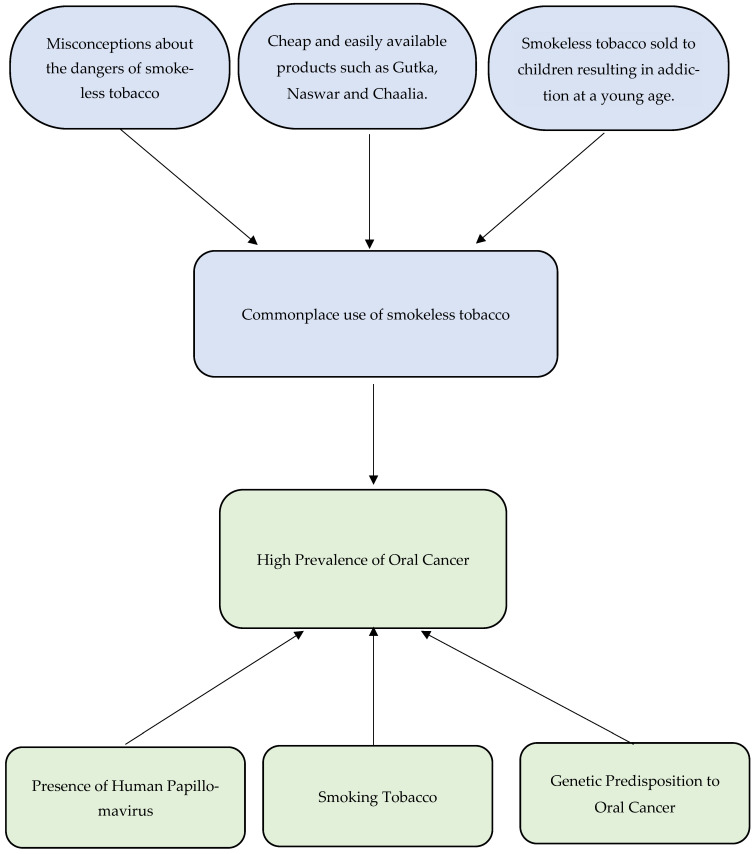
Flowchart demonstrating significant themes.

The themes developed during this review are demonstrated in Figure 2.

## 4. Discussion

Oral cancer is one of the most important public health concerns faced by Pakistan today and poses a threat to the well-being and productivity of a population that is already crippled by a destabilised political environment and debilitating economy. It is of the utmost importance to identify all contributing factors and take preventive measures to curb the spread of this disease. As with other healthcare crises, the high prevalence of OC in the country is affected by the unique cultural and social circumstances in which the individuals reside.

This review found that smokeless tobacco is the most prominent risk factor for oral cancer in the country. Different types of chewing tobacco are widely accessible in the nation, ranging from loose to finely cut and shredded leaves, while snuff can be acquired in the form of ground tobacco, which is available in either dry or moist sachets. These various ST products are given different names depending on the region and may be known as supari, gutka, mawa, qiwam, mainpuri, zarda, and naswar [19]. Other studies have found gutka to be more addictive compared to other ST products and it is also a first step to other harmful products.

In Pakistan’s neighbouring country, India, the same issue is prevalent. It has been reported that up to five million children below the age of 15 years have developed an addiction to gutka [33]. Zhao et al. [34] found that use of SLT was a concerning issue in the subcontinent, and approximately 231 million adults at the age of 15 years or above used ST in Bangladesh, India, and Pakistan, comprising 40.3% to 74.7% of the overall tobacco use. The high use of smokeless tobacco may be due to the misconception that chewing tobacco is less dangerous than smoking. In addition, it is readily sold to children who may develop an addiction to the substance at a very young age. It is paramount that more awareness be spread about the many dangers of ST products and that policy changes be undertaken to control their sale.

An important aim of this review was to look at studies in the region that targeted the less-discussed risk factors such as human papillomavirus infection and genetic predisposition. Several included studies suggest that Human Papillomavirus may be a significant contributor to the OC load in Pakistan. For instance, Zil-E-Rubab et al. [17] found that patients infected with HPV 16/18 had significantly greater odds of developing OC compared to patients who did not have this virus. Furthermore, patients infected with multiple strains of the high-risk virus possess a still greater risk of suffering from the malignancy [17,18].

Baig et al. [31] reported that consistent exposure to gutka may increase the frequency of the Human Papillomavirus. The presence of multiple risk factors such as poor oral hygiene, tobacco use, and HPV infection may act synergistically to increase the chances of developing a malignancy [18]. These findings are consistent with studies conducted in other countries on the association between HPV and oral cancer. A systematic review conducted by Mohammed et al. [35], examining the association between the virus and oral cancer, reported that the high-risk strains of HPV can significantly contribute to the development of oral carcinomas.

Only one study [28] failed to find any association between HPV and oral cancer. All 58 subjects included in the study were negative for HPV, and the authors suggest that the correlation between the virus and the malignancy could be overstated, especially in Pakistan. However, this discrepancy could have arisen due to the small sample size and may not be relevant to the whole population. Overall, very little research has been conducted to establish the role of this virus on the oral cancer burden in Pakistan. It is suggested that larger, more conclusive studies be conducted.

Tobacco smoking is a known risk factor for OC, and this review indicates that there is a significant need for addressing this issue in Pakistan. A study by Junaid et al. [32] reported that out of the 200 patients with proven malignancy of the oral cavity, 61.5% of them were cigarette smokers. Another study conducted in Sindh [14] found that the mixture of smoking and alcohol usage constituted a high risk for oral cancer. This is in line with other studies conducted in the region and all over the world.

Muwonge et al. [36] found that in India, alcohol can act synergistically with smoking and raises the risk of developing oral carcinomas by 10- to 15-fold. It is important to consider the multiple factors that go into the use of cigarettes in Pakistan. Hameed and Malik [37] stated that a predominant 69.8% of smokers came from middle-class backgrounds and 71.3% were wholly unaware of any alternative product. A majority (68.2%) of smokers reported that they were keen to quit but could not because of nicotine addiction. Lack of smoking cessation services may thus be a major factor in the ongoing fight against the tobacco epidemic. It is of the utmost importance that in conjunction with spreading awareness about smoking and the various life-threatening diseases it can cause, i.e., lung cancer and oral cancer, there is also a need for tobacco harm reduction products, therapeutic services, and emotional support.

Genetic predisposition can be considered a significant contributor to the development of OSCC [33]. This explains the commonly observed phenomenon that many habitual life-long tobacco users do not develop the malignancy. Some individuals may inherit a susceptibility that results in an impaired ability to metabolise various carcinogens and/or a decreased ability to repair any DNA damage that occurs from tobacco use [38]. This review discovered that too few studies have been conducted on the genetic predisposition of the Pakistani population to the risk of developing oral malignancies. The studies that are included in this review suggest that genetic predisposition to oral cancer may play a significant role in the causation. For instance, Alamgir et al. [25] reported that when CYP1AQ homozygous and GSTM1 null variants occur simultaneously in individuals, the risk of developing the disease increases over 12-fold.

Another factor to consider is that Pakistan is a multi-ethnic country, and the distribution of tobacco-metabolising enzymes also varies significantly among different populations [26]. More research needs to be conducted on the genetic predilection of Pakistanis to the disease and oral surgeons should practice extreme vigilance. No lesion should be ignored, and familial history should be a significant part of any preliminary diagnosis. Furthermore, awareness should be spread amongst the population about the many risk factors for oral cancer and the necessary preventive measures.

### 4.1. Strengths and Limitations

#### 4.1.1. Strengths

One of the major strengths of this systematic review is its reliability and validity. By providing a rigorous and comprehensive synthesis of all available evidence on the risk factors for oral cancer in Pakistan, it ensures a thorough examination of multiple studies, enhancing the reliability and validity of the findings. Another important strength is reproducibility, which is ensured using established guidelines such as PRISMA, enabling transparency and replication of the study, and allowing other researchers to verify the results and conclusions. This study has pooled data from multiple studies conducted in Pakistan, and this has enabled it to provide a clear picture of the research conducted on the risk factors for oral cancer in the country.

#### 4.1.2. Limitations

This systematic review may be susceptible to some degree of publication bias, whereby studies with significant findings are more likely to be published, leading to an overrepresentation of certain risk factors. As Pakistan has an extremely unique profile, the findings of this study may apply to the population of the country, but it might have limited value elsewhere. Applying it to other locations with different risk factors, socioeconomic profiles, and healthcare systems should be carried out with caution. Moreover, the full text for some studies could not be obtained, which could have provided some important findings and conclusions.

## 5. Conclusions and Recommendations

This review indicates that more research needs to be conducted on the risk factors for oral cancer, particularly genetic predilection, and HPV virus. Tobacco chewing in all its forms such as gutka, naswar, and manipuri needs to be curbed through policy changes, strict prohibition of sale to underage individuals, increased taxes, and education. Nicotine addiction is a significant issue in Pakistan, and sufferers do not have access to or knowledge about safer options. The authors recommend that safer and cheaper tobacco alternatives be introduced, smoking cessation centres be established, and more information be spread about how to counter the addiction. In addition, awareness about HPV and its relation to OC needs to be disseminated, and healthy sexual behaviours need to be encouraged.

Oral healthcare professionals need to show more vigilance to promote early diagnosis, and patients should be encouraged to increase routine checkups. Patients exposed to one or multiple risk factors should be educated and appointed for regular examinations.

Furthermore, studies need to be conducted on sociodemographic and economic factors that can lead to the formation of harmful habits. Most research has only been conducted in major cities and capitals, and there is a need for more representation from remote areas. Areas that are less easily accessible and, consequently, less studied, should be focused on. In conclusion, oral cancer is one of the most important public health issues in the country, and a holistic preventative approach is required to curb its incidence.

## Figures and Tables

**Table 1:** PECOS.

P—Population	Individuals 18 years of age or older suffering from malignancy of the lip or oral cavity, residing in Pakistan.
E—Exposure	The various risk factors or predisposing factors of cancer of the oral cavity or lip.
C—Comparison	No specific comparison needed for risk factor analysis.
O—Outcome	Identification and understanding of risk factors associated with oral cancer in Pakistan
S—Study Design	Peer-reviewed reports of observational studies and clinical trials will be included in this study.

**Table 2:** Inclusion and Exclusion Criteria.

	Inclusion Criteria	Exclusion Criteria
Population	Individuals 18 years of age or older suffering from malignancy of the lip or oral cavity, residing in Pakistan.	Individuals younger than 18, patients suffering from head and neck cancers other than oral cancers.
Exposure	The various risk factors or predisposing factors for cancer of the oral cavity or lip.	Individuals who use known carcinogen-containing products but do not suffer from oral cancer were excluded.
Comparison	No specific comparison group needed.	
Outcome	Identification and understanding of risk factors associated with oral cancer in Pakistan.	Studies that do not contribute to the identification and understanding of risk factors associated with OC were excluded.
Study Design	Peer-reviewed reports of observational studies and clinical trials published in English language were included in this study.	Any secondary studies were excluded from this study. Any studies not published in English language were excluded.

**Table 7:** Data extraction table (characteristics of the 20 included studies and summary of findings).

Reference	Study Design and Methods	Context	Sample Size	Is Aim Specific to Risk Factors for OC?	Aim of the Study	Key Findings Regarding Risk Factors for OC	Limitations
Awan et al. (2016) [15]	Case–control study	Karachi, Pakistan	268	Yes	To evaluate the risk of oral cancer associated with gutka and other ST products.	Gutka most used product leading to 1/3rd of cases. Chewing tobacco was 5.32 times higher compared to controls.	Controls may not disclose information about tobacco-chewing habits.
Alamgir et al. (2022) [25]	Cross-sectional case–control study	Karachi, Pakistan	238	Yes	Discover the role played by molecular mechanisms in OSCC carcinogenesis in the target population.	High risk of OSCC with the presence of combined gene polymorphisms of phase 1 and phase 2 enzymes.	Lack of representation of certain categories of tobacco consumption.
Alamgir et al. (2022) [26]	Cross-sectional, case–control study	Karachi, Pakistan	358	No	To evaluate intra-ethnic variability of CYP1A1-Mspl, GSTM1-Null, and GSTT1-null metabolic gene polymorphisms.	CYP1A1 Mspl m1/m2 and m2/m2 polymorphisms found in 85.7% of OSCC cases.	Too few members of Pushto-speaking community. Lack of representation of certain genotypes.
Zakiullah et al. (2015) [10]	Case–control study	Khyber Pukhtoonkhwa, Pakistan	351	Yes	To evaluate the potential role of CYP1AQ, GSTM1, and GSTT1 gene polymorphisms in the susceptibility to OC in the Pashtun Population of KPK.	Null genotypes of both GST genes with almost 3-fold higher risk of OC compared to wild type.	Since the subjects of the study were limited to the Pashtun population, findings may not be generalizable. Less controls as opposed to cases.
Alamgir et al. (2016) [30]	Retrospective observational study	Karachi, Pakistan.	150	Yes	Analysis of tumour characteristics and their association with common risk factors.	Habit of tobacco chewing found in approximately 78% of cases.	Dependent on the quality and accuracy of historical medical records.
Masood et al. (2011) [16]	Case–control study	Islamabad, Pakistan	378	Yes	Genetic changes in CYP1A1, GSTM1, GSTT1, and GSTP1 genes and their association with risk of OC.	Significantly higher proportion of OC patients had GSTM1 deletion genotype as opposed to controls.	Matching of controls by age and sex might not eliminate all potential confounding factors such as smoking, diet, etc.
Mohiuddin et al. (2016) [29]	Multi-centre cross-sectional study	Karachi, Pakistan	1774	Yes	To determine the relationship between age, gender, and other associated risk factors linked with the malignant transformation of OSMF into OSCC.	Females included in the study showed higher malignant transformation than males.	Uses non-probability convenience sampling to select participants which may cause sampling bias.
Zil-E-Rubab et al. (2018) [17]	Case–control study	Karachi, Pakistan	300	Yes	To find out the association between HPV 16/18 genotypes in Pakistan patients with OSCC.	Pts infected with HPV 16/18 had significantly higher chances of developing OSCC as opposed to patients who did not have high-risk strains.	Only 100 cases as opposed to 200 controls.
Baig et al. (2012) [31]	Descriptive study	Karachi, Pakistan	262	No	To determine the frequency of HPV in eaters of Gurka presenting with oral lesions.	Significantly higher frequency of high-risk HPV strains in subjects who have been chewing tobacco for over 10 years.	Most of the participants were male; this was a single-centre study, limiting generalizability.
Sarwar et al. (2022) [18]	Cross-sectional study	Different hospitals of Pakistan in Islamabad and KPK.	152	No	To detect high-risk genotypes of HPV and protein expression of NF-κB signalling pathway in HNC patients with HPV infection.	Strong association between HR-HPV infection and oral cavity cancer patients	Cross-sectional design unable to inform causality.
Junaid et al. (2019) [32]	Descriptive retrospective study	Islamabad, Pakistan	200	No	To determine the frequency of carcinoma of various oral cavity subsites along with risk factors like smoking.	Tobacco-smoking found to be the major risk factor for a higher frequency of oral cancer. No such association was found for age and gender.	Retrospective design inherently prone to some degree of recording problems, which could skew the data in one way or another, making the findings less reliable.
Alamgir & Shaikh. (2021) [27]	Cross-sectional case–control study	Karachi, Pakistan	358	No	To find out the level of exposure of oral mucosa to tobacco products in terms of lifetime tobacco indices.	83.4% of the patients suffering from OC were found to be tobacco users in one way or another.	Non-availability of histopathology slides for oral pre-cancerous lesions.
Khan et al. (2017) [19]	Multi-centre case–control study	Khyber-Pukhtoonkhwa, Pakistan	258	Yes	Assess the association between naswar and the risk of OC.	Ever and current users of naswar had a 20-fold higher risk of oral cancer as opposed to non-users.	Study sample, particularly hospital controls, may not be representative of the general population of KPK.
Merchant et al. (2000) [20]	Case–control study	Karachi, Pakistan	228	Yes	To clarify the independent association between paan and oral cancer.	People using paan w/o tobacco were 9.9 times more likely to develop OC, and people using paan with tobacco were 8.4 times more likely to develop OC.	Few cases compared to controls, plus some degree of recall bias.
Shahid et al. (2018) [21]	Case–control	Karachi, Pakistan	234	No	To obtain and compare comprehensive metabolic profiles of plasma samples of pure tobacco snuff dippers with oral cancer.	Strong correlation between regular tobacco snuff dippers and oral cancer.	-
Naqvi et al. (2020) [28]	Cross-sectional study	Karachi, Pakistan	58	Yes	To find out frequencies of EBV, CMV, and HPV infection among patients with OSCC in the Pakistani population.	All cases negative for HPV; CMV detected in 5 percent of cases and 25.86% of cases were positive for EBV.	Very small sample size.
Mugheri et al. (2018) [14]	Case–control study	Hyderabad and adjoining areas of Sindh	662	Yes	To estimate the association of various epidemiological risk factors for oral cancer.	Alcohol, cigarette smoking, use of khula ghee and pakwan, Manipuri, and collective addictions are significant risks for oral cancer.	Recall bias i.e., patients with oral cancer are more likely to mention causative factors than controls.
Azhar et al. (2018) [22]	Case–control study	Karachi, Pakistan	124	Yes	To ascertain prevalent risk factors for OC in the Pakistani population.	Smokeless tobacco found to be an independent risk factor for oral cancer.	Small sample size + recall bias.
Khan et al. (2020) [23]	Case–control study	Lahore, Pakistan	210	Yes	To evaluate the risk of oral cavity cancer with the use of various smokeless tobacco products.	Positive association between ever users of smokeless tobacco and risk of OC	Less proportion of female patients and most of the patients were derived from one hospital which indicates limited generalizability.
Mehdi et al. (2019) [24]	Case–control study	Hospital-setting, Karachi, Pakistan	148	Yes	To find an association between survivin polymorphism and the prevalence of OSCC in a subset of the Pakistani population.	Significant association between CC and GG genotypes of survivin and OSCC prevalence.	Too few cases in the sample and hospital settings indicate little generalizability.
